# The Matron's Department

**Published:** 1905-09-09

**Authors:** 


### HOSPITAL ADMINISTRATION.

### CONSTRUCTION AND ECONOMICS.

### THE MATRON'S DEPARTMENT.

#### IV. THE WARD STAFF.

In illustration of the variety of hospital administration we cannot do better than give a few examples of ward equipment in some typical hospitals in town and country from information courteously supplied by the heads of these institutions.

The average of beds which falls to the staff has been estimated by omitting all nurses employed exclusively outside the wards, including, however, the extra nurses required for leave, holidays, and emergencies.

*St. George's Hospital.*

| | Sister. | Staff Nurse. | Probationers. |
|---|---|---|---|
| 3 wards 9 beds each ... (day) | 1 | 1 | 1 |
| " " ... (night) | — | 1 | 1 |
| 5 wards, 12–14 beds each (day) | 1 | 1 | 1 |
| " " (night) | — | 1 | ½ |
| 8 wards, 23–26 beds each (day) | 1 | 1 | 3 |
| " " (night) | — | 1 | 1 |
| 2 wards, 33–35 beds each (day) | 1 | 1 | 5 |
| " " (night) | — | 1 | 2 |

Average of beds divided among day and night staff, 2.
Proportion of first year probationers, 21 per cent.

*King's College Hospital.*

| | Sister. | Staff Nurse. | Probationers 1st and 2nd year. |
|---|---|---|---|
| Ward of 30 beds ... (day) | 1 | 1 | 4 |
| " " ... (night) | — | 1 | 1 |

This allows of four consecutive hours off duty for each nurse.
Average of beds among day and night staff, 2·4.

*Royal Free Hospital.*

| | Sister. | Staff Nurse. | Senior Prob. | Junior Prob. |
|---|---|---|---|---|
| Full-sized ward ... (day) | 1 | 1 | 1 | 1 |
| " " (night) | One night nurse. | | | |

Average of beds among day and night staff, 3·1.
Proportion of first year probationers, 24 per cent.

*Seamen's Hospital.*

The wards are all small, containing from 3 to 7 beds. They are grouped together in floors, and the system in practice works out at an extreme economy of nursing.

| | Sister. | Staff Nurse. | Probationers 1st and 2nd year. |
|---|---|---|---|
| Surgical floor of 53 beds (day) | 1 | 2 | 2 |
| " " (night) | — | 1 | 1 |
| Accident floor, 42 beds and 6 cots ... ... (day) | 1 | 2 | 3 |
| " " ... (night) | — | 1 | 1 |
| Medical floor, 41 beds (day) | 1 | 2 | 3 |
| " " (night) | — | 1 | 1 |

Average of beds among day and night staff, 5·3.
Proportion of first year probationers, 39 per cent.

*Great Northern Central.*

| | Sister. | Staff Nurse. | Senior Prob. | Junior Prob. |
|---|---|---|---|---|
| Ward of 25 Beds (day) ... | 1 | 1 | 1 | 1 |
| " " (night)... | One night nurse. | | | |

Average of beds among day and night staff 3·7.
Proportion of first year probationers 25 per cent.

*Addenbrooke's Hospital, Cambridge.*

| | Sister. | Staff Nurse. | Senior Prob. | Junior Prob. |
|---|---|---|---|---|
| Surgical wards, beds 27–32 ... ... ... | 1 | 1 day and 1 night. | 1 | 2 |
| Medical wards, beds 26... | 1 | 2 | 1 | 1 |
| Children, 21 beds ... | 1 | 2 | (1 day and 1 night.) | 2 |

Average of beds among day and night staff 2·3.
Proportion of first year probationers, 26 per cent.

*Sunderland Infirmary.*

Ten of the wards contain 12 beds each; four have 17 beds, one has 14. There are three small wards with six beds.

| | Sister. | Staff Nurse. | Prob. | Male Attendant. |
|---|---|---|---|---|
| Female surgical, 2 wards, 26 beds | 1 | 2 | 2 | — |
| " " " (night) | — | 1 | — | — |
| Male surgical, 4 wards, 48 beds | 1 | 4 | — | — |
| " " " (night) | one nurse and one male attendant. | | | |
| Medical (2 male 2 female) 4 wards, 48 beds | 1 | 4 | 2 | 2 |
| " " " (night) | one nurse and one male attendant. | | | |
| Children's (1 med., 1 surg.) 2 wards, 34 beds | 1 | 2 | 4 | — |
| (night) | one nurse. | | | |

Average of beds among day and night staff, 5.
Proportion of first year probationers, 23 per cent.

*Sussex County Hospital.*

| | Sister. | Nurses and Probationers. |
|---|---|---|
| Large ward, 31 beds (day) ... | 1 | 4 |
| " " (night) ... | — | 2 |
| Smaller wards ... (day) ... | 1 | 3 |
| " " (night) ... | — | 2 |

Average of beds among day and night staff, 3·2.
Proportion of first year probationers, 20 per cent.

*Western Infirmary, Glasgow.*

| | Sister. | Staff Nurse. | Senior Prob. | Junior Prob. |
|---|---|---|---|---|
| Full-sized wards (in cubicles) (day) | 1 | 1 | 2 | 2 |
| " " " (night) | — | — | 2 | 2 |
| Heavy surgical wards ... (day) | 1 | 2 | 2 | 3 |

Average of beds among day and night staff, 3·1.
Proportion of first year probationers, 26 per cent.

*Royal Infirmary, Glasgow.*

In this hospital there are no sisters and their place is supplied by "head nurses." The wards are irregular in size.

| | Head Nurse. | Assistant Nurses. | Probationers. |
|---|---|---|---|
| Ward of 26 beds (day) ... | 1 | 2 | 1 |
| " " (night) ... | — | 1 | 1 |
| Ward of 13 beds (day) ... | 1 | 1 | 1 |
| " " (night) | one night nurse. | | |

Average of beds among day and night 3·8.
Proportion of first year probationers, 19 per cent.

It will be seen that with one or two exceptions the variations in number takes place among the probationer class. The sister, the staff nurse or third-year probationer, and two probationers in their first and second years, form the groundwork of all the systems for full-sized wards containing 25 beds and over. Where much teaching is undertaken the number of probationers swells to four.

Irregularities of construction may justifiably give rise, as we have already pointed out, to an increase in the number of the staff, but this may also prove an occasion of triumph to the resourceful administrator. The latter result is illustrated in the averages of the Seamen's Hospital. Exceptionally able sisters are required for the superintendence of a floor of from 40 to 50 beds, separated up into eight or ten wards, and the high character of the nursing at this hospital shows unmistakably that such sisters are secured and retained in its service.

It is not possible, in the face of the varied averages quoted in the preceding article, to lay down any hard and fast rule as to the correct proportions of the nursing staff in a general hospital. There are, however, so many instances in which the nursing is of the highest order on the basis of 3 or 3·5 beds to the ward staff that it should be in the matron's power, where this proportion of nurses is exceeded, to account for the excess.

The work may be of an exceptionally arduous character, as is the case in the Middlesex Hospital Cancer Charity, or the building may be in process of reconstruction, as in the case of the London Hospital, or the wards may be ill-planned, as in the case of Guy's Hospital, or there may be an exceptionally valuable training-school, as in the case of King's College Hospital. But whatever the cause may be it should be clearly understood by all the authorities that the normal average of nurses sufficient under ordinary conditions for securing a high standard of nursing without undue strain on the nurses has been over-passed.

